# Stroke as an Unusual First Presentation of Lyme Disease

**DOI:** 10.1155/2015/389081

**Published:** 2015-12-16

**Authors:** Mohamad Almoussa, Angelika Goertzen, Barbara Fauser, Christoph W. Zimmermann

**Affiliations:** Department of Neurology, St. Josef Hospital, The Academic Hospital of Duisburg-Essen University, Mülheimer Strasse 83, 46045 Oberhausen, Germany

## Abstract

*Introduction*. Lyme neuroborreliosis is a nervous system infection caused by spirochete* Borrelia burgdorferi* with diverse neurological complications. Stroke due to cerebral vasculitis is a rare consequence of neuroborreliosis and has been described in just a few case reports.* Case Presentation*. Here, we report the case of a 43-year-old patient who presented with discrete left-sided hemiparesis and amnestic cognitive impairment. Brain magnetic resonance imaging showed a thalamic infarct, and serological and cerebrospinal fluid (CSF) tests confirmed the diagnosis of active neuroborreliosis. The antibiotic treatment with intravenous ceftriaxone for three weeks led to an improvement of the symptoms and remarkable regression of radiological findings, but not to full recovery of the amnestic cognitive disorder.* Conclusion*. Lyme neuroborreliosis should be suspected in patients with cerebrovascular events without obvious risk factors, especially those living in endemic areas such as northern Europe or those who have been exposed to ticks and those with clinical or radiological findings suggesting Lyme neuroborreliosis, in order to establish the diagnosis and start a proper antibiotic therapy.

## 1. Background

Lyme borreliosis is a multisystem infection caused by the spirochete* Borrelia burgdorferi sensu lato* species, which are transmitted by the bite of infected* Ixodes ricinus* ticks. While* Borrelia burgdorferi sensu stricto* is the sole pathogen in North America, five different species, most often* Borrelia afzelii* and* Borrelia garinii*, can cause the disease in Europe and Asia.

Lyme borreliosis is an endemic widespread in the northern global hemisphere. In Germany, an analysis of health insurance data showed an annual incidence of 261 per 100,000 [1, 2]. The clinical manifestations of Lyme disease are diverse and vary with its stage, from erythema migrans (EM) in early localized stage to neurologic and/or cardiac complications in the early disseminated stage. Late Lyme disease occurs months to a few years after the primary infection and is typically associated with arthritis and/or neurologic problems. Acrodermatitis chronica atrophicans is a cutaneous manifestation of late Lyme disease. However, some patients may present in later stage without any signs or symptoms of earlier Lyme disease [3]. Moreover, nonspecific symptoms, including fatigue, anorexia, headache, neck stiffness, and myalgias, may appear in each stage. This wide variety of clinical manifestations is partly due to differences in the infecting species of the bacterium and can lead to a late diagnosis of the disease [4]. The involvement of the central or peripheral nervous system secondary to systemic infection with* Borrelia* bacterium is called Lyme neuroborreliosis (LNB). Approximately, 13% of patients with Lyme disease develop neurological complications such as meningitis, meningoencephalitis, facial palsy, cranial neuritis and radiculoplexitis, rarely stroke, intracerebral hemorrhage, and sinus thrombosis [2, 5–10].

Herein, we report cerebral ischemic stroke as a rare and an unusual first manifestation of LNB.

## 2. Case Presentation

A previously healthy 43-year-old man, without any cardiovascular risk factors, presented in a bad general physical condition with a 2-week history of malaise, headache, and amnestic cognitive impairment. The patient could recall a tick bite four months ago during holiday in Netherlands but could not recall having an EM. Routine laboratory investigations showed no abnormalities.

On physical examination, he had discrete left-sided hemiparesis, and he was fully oriented but showed massively slowed movements and slept during the interview. There were a marked short-term memory loss, psychomotor impairment, and a mildly stiff neck as a sign of meningitis.

**Figure 1:**
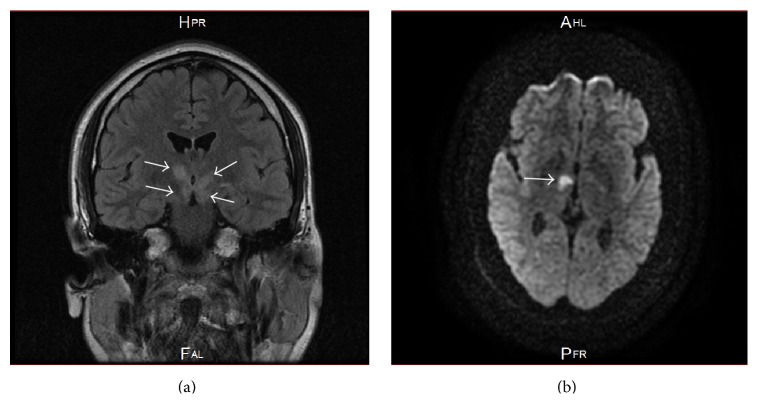
Images of the brain MRI study. (a) Coronal FLAIR-T2 showing bilateral pathological hyperintense signal abnormalities in periventricular, periaqueductal area, in both crura cerebri, and in both hypothalami (arrows). (b) Axial diffusion weighted imaging DWI showing a right thalamic infarct as a hyperintense signal.

The brain magnetic resonance imaging (MRI) revealed a right thalamic infarct as a hyperintense signal on the diffusion weighted imaging with correspondent low signal on the ADC-Map and pathological hyperintense signal abnormalities on FLAIR/T2 in periventricular, periaqueductal area, in both crura cerebri, and in both hypothalami (Figure 1).

The CSF examination showed notable inflammatory changes and a substantially disturbed blood brain barrier (pleocytosis 43 cells/*μ*L; 94% lymphocytes; Albumin 588 mg/L), combined with intrathecal synthesis of IgG 49%, IgA 38%, and IgM 81% (*n* < 10%). The* Borrelia burgdorferi* antibody index (AI) was elevated for IgG = 38.5 and IgM = 5.9 (*n* = 0.6–1.5). The immunoblot analysis identified the following antigens of* Borrelia burgdorferi* in the CSF and serum: VlsE, pG, p83, and BBO323.

Further laboratory investigations for protein C and protein S activity, complement components C3c and C4, and anti-cardiolipin and anti-beta2-glycoprotein antibodies were normal. The antinuclear antibodies, antineutrophil cytoplasmic antibodies, anti-DNA antibodies, antiextractable nuclear antigens, and hepatitis B and C serology were also negative.

The transesophageal echocardiogram (TEE), stroke-care monitoring, and carotid Duplex ultrasonography excluded any cardiac or arterial source of embolism.

**Figure 2:**
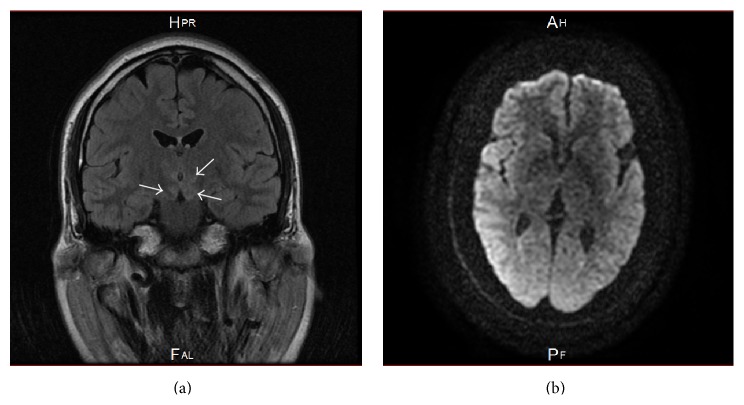
Brain MRI scans ten days after initiating the antibiotic therapy showing residual hyperintense signal alternations in the hypothalamus, in the left crura cerebri, and in the left thalamus (arrows) on the coronal FLAIR-T2 in (a) and complete regression of the signal abnormality on the axial diffusion weighted imaging DWI in (b).

We started a 3-week course of intravenous ceftriaxone at a dosage of 2 g daily according to the European Federation of the Neurological Societies (EFNS) guidelines [11]. Cranial MRI scans during the antibiotic treatment revealed remarkable resolve of the signal abnormalities (Figure 2); in correlation, quick normalization of the left-sided hemiparesis and the psychomotor impairment could be noticed. Unfortunately, the cognitive amnestic impairment did not improve and the patient was discharged to a stationary cognitive rehabilitation therapy.

## 3. Discussion

Case reports describing stroke due to LNB are rare. Our case was shown to be consistent with other observations that stroke occurs with LNB when associated with pleocytosis and meningitis [9, 10]. The serological and cerebrospinal fluid (CSF) tests confirmed the diagnosis of active neuroborreliosis. Cranial MRI scans demonstrated a right thalamic infarct with multiple bilateral vascular changes supporting the notion that the common location of stroke in the setting of LNB is in the posterior circulation [12].

The pathophysiology of stroke in the context of LNB has not been fully understood yet. Localized vasculitis was the most likely proposed mechanism of stroke in the setting of LNB. Angiography and transcranial Doppler have already demonstrated segmental stenosis of cerebral arteries compatible with vasculitis [13, 14]. Although Duplex ultrasonography was unremarkable in the present case, this does not exclude the local vasculitis, since changes in small cerebral vessels cannot be shown in Duplex ultrasonography. However, we could see bilateral vascular changes in cranial MRI scans; furthermore, no clinical or laboratory evidence for systemic vasculitis was present.

Despite the quick radiological and clinical response to antibiotic therapy, a mild short-term memory loss was still persistent at the follow-up visit two months later.

## 4. Conclusion

Infection with* Borrelia burgdorferi* should be considered in stroke patients without obvious risk factors, especially those who live in endemic areas such as northern Europe or those exposed to ticks, and in patients with clinical or radiological findings suggesting the infection with* Borrelia burgdorferi*, in order to establish the diagnosis and start an efficient antibiotic therapy before irreversible complications may occur.

## Abbreviations

MRI: Magnetic resonance imaging
CSF: Cerebrospinal fluid
LNB: Lyme neuroborreliosis
AI: Antibody index.
